# COVID‐19–Associated Reversible Cerebral Vasoconstriction Syndrome: A Rare Case of Secondary Thunderclap Headache

**DOI:** 10.1155/crnm/4136897

**Published:** 2026-03-16

**Authors:** Seraj Makkawi, Osama Khojah, Mohammed Alsalmi, Adilah Alturaifi, Bader Ibrahim, Abdulrahman Alshamy

**Affiliations:** ^1^ College of Medicine, King Saud bin Abdulaziz University for Health Sciences, Jeddah, Saudi Arabia, ksau-hs.edu.sa; ^2^ King Abdullah International Medical Research Center, Jeddah, Saudi Arabia, kaimrc.med.sa; ^3^ Department of Neurology, Ministry of the National Guard-Health Affairs, Jeddah, Saudi Arabia; ^4^ Department of Medical Imaging, Ministry of the National Guard-Health Affairs, Jeddah, Saudi Arabia

**Keywords:** case report, COVID-19, RCVS, reversible cerebral vasoconstriction syndrome, SARS-CoV-2

## Abstract

Reversible cerebral vasoconstriction syndrome (RCVS) is a condition characterized by severe, sudden headaches, and transient cerebral artery constriction, often triggered by medications, physical activities, or comorbidities. Recent studies have linked RCVS to SARS‐CoV‐2 infection. This case report presents a novel instance of RCVS with nonaneurysmal subarachnoid hemorrhage (SAH) following COVID‐19. A 44‐year‐old female with COVID‐19 presented with a sudden, severe headache, confusion, dizziness, and photophobia. Imaging revealed extensive SAH and hydrocephalus. Despite the absence of classic RCVS triggers, a cerebral angiogram showed multifocal vasospasms in both anterior and posterior circulations which later resolved on repeat cerebral angiography. The patient’s condition improved with external ventricular drainage followed by symptomatic treatment. She was diagnosed with RCVS after achieving an RCVS_2_ score of seven points. She had a final modified Rankin scale of zero at 40‐month follow‐up. This case highlights the potential association between COVID‐19 and RCVS, even in the absence of common triggers. While causality cannot be definitively established, the temporal relationship and plausible biological mechanisms warrant further investigation. This report underscores the importance of considering RCVS in patients with neurological symptoms following COVID‐19 and contributes to the understanding of the virus’s impact on the vasculature.

## 1. Introduction

Coronavirus disease 2019 (COVID‐19) is an infectious disease that results from the severe acute respiratory syndrome Coronavirus 2 (SARS‐CoV‐2). Since late 2019, the global community has grappled with a pandemic caused by SARS‐CoV‐2, resulting in significant morbidity and mortality worldwide. Despite the World Health Organization’s declaration of the pandemic’s end, COVID‐19 and its associated sequelae persist [1]. SARS‐CoV‐2 commonly affects the respiratory system but has been reported to affect many body organs including the brain and blood vessels [2]. The neurologic manifestations associated with COVID‐19 included stroke, Guillain–Barré syndrome, posterior reversible encephalopathy syndrome (PRES), and reversible cerebral vasoconstriction syndrome (RCVS) [3].

RCVS is a clinical and radiological disorder defined by severe “thunderclap” headaches, which may be accompanied by the presence of neurological symptoms. It is distinguished by cerebral artery (CTA) constriction visible on imaging which classically resolves within 3 months [3]. RCVS has been associated with certain triggers such as medications (pseudoephedrine, triptans, oral contraceptive medications, selective serotonin or serotonin–norepinephrine reuptake inhibitors, or amphetamines), activities (cough, sexual intercourse, or vigorous exercise), and medical comorbidities (migraines, systemic lupus erythematosus, carotid, or vertebral artery dissections) [4]. RCVS has also been linked to SARS‐CoV‐2 infections and COVID‐19 vaccination [5]. RCVS is clinically important because it may be complicated by ischemic stroke, intracerebral hemorrhage, or subarachnoid hemorrhage (SAH), leading to significant morbidity. In this article, we present a case of RCVS with nonaneurysmal SAH following SARS‐CoV‐2 infection, highlighting the clinical and radiological features, diagnostic approach, and long‐term outcomes. This case expands the limited literature on COVID‐19–associated RCVS by providing long‐term follow‐up, thereby contributing to characterization of disease evolution and outcomes. It is notable for the close temporal association between COVID‐19 and symptom onset in the absence of traditional RCVS triggers, supported by an extensive diagnostic evaluation confirming fulfillment of established RCVS criteria.

## 2. Case Presentation

**FIGURE 1:**
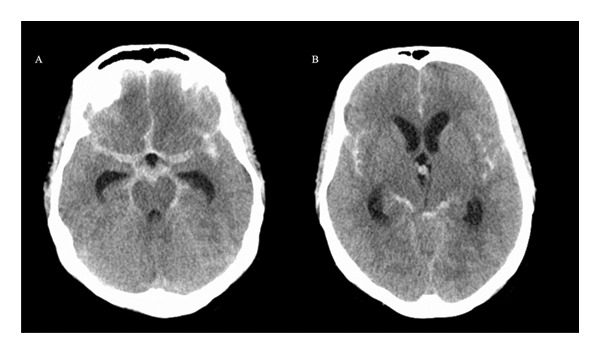
(A, B) Noncontrast axial brain CT performed on Day 1. (A) Extensive subarachnoid hemorrhage (SAH) within the basal cisterns and a distended temporal horn of the lateral ventricle. (B) SAH within the basal cisterns and bilateral sylvian fissures, and distended frontal and occipital horns of the lateral ventricle.

A 44‐year‐old female presented to the emergency department for a sudden‐onset severe headache which reached maximal intensity in a few seconds described as the worst headache in her life with a poor response to simple analgesia. This was associated with confusion, dizziness, photophobia, and phonophobia. Aside from symptoms of an upper respiratory tract infection due to PCR‐confirmed COVID‐19 that began 10 days before presentation, her medical history was unremarkable. She was not on any regular medications or taking symptomatic treatments for the COVID‐19 infection. Examination showed a vitally stable, normotensive, patient disoriented to time with a Glasgow Coma Scale (GCS) of 14/15 and unremarkable cranial nerves, motor, sensory, and coordination functions. Computed tomography (CT) of the brain showed extensive SAH with hydrocephalus (Figure 1); she underwent emergency insertion of an external ventricular drain (EVD).

**FIGURE 2:**
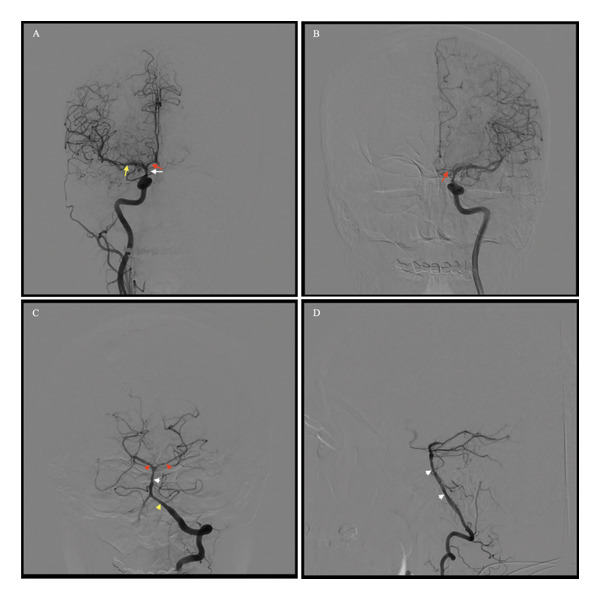
Cerebral angiogram performed on Day 12. (A) AP view of the right anterior circulation demonstrates narrowing of the supraclinoid segment of the internal carotid artery (white arrow), the M1 segment of the middle cerebral artery (yellow arrow), and the A1 segment of the anterior cerebral artery (ACA) (red arrow). (B) AP view of the right anterior circulation demonstrates narrowing of the A1 segment of the ACA (red arrow). (C) AP view of the left posterior circulation demonstrates narrowing of the left vertebral artery (yellow arrowhead), basilar artery (white arrowhead), and bilateral posterior cerebral arteries (red arrowhead). (D) Lateral view of the left posterior circulation demonstrates narrowing of the basilar artery (white arrowhead). No vascular malformations, aneurysms, intimal flaps, dilated segments, occlusions, or other structural abnormalities were identified.

CT angiography of the CTA did not reveal a vascular pathology. Conventional cerebral angiogram showed evidence of string of beads as multifocal vasospasms of the vessels of the anterior and posterior circulation without evidence of aneurysms and vascular malformations (Figure 2).

**FIGURE 3:**
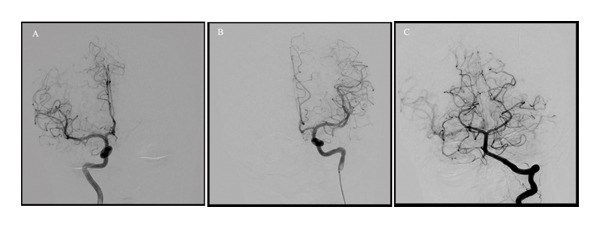
Cerebral angiogram performed 9 months from initial presentation. (A) AP angiography of the right anterior circulation, (B) AP angiography of the left anterior circulation, and (C) AP angiography of the left posterior circulation demonstrated a normal and smooth caliber of the cerebral vasculature without any obvious irregularities.

Even though her GCS improved to 15/15 after the EVD insertion, she was transferred to our institution (King Abdulaziz Medical City, Jeddah, Saudi Arabia), a tertiary care center, for further investigation, neurosurgical evaluation, and neurocritical observation. Cerebrospinal fluid analysis showed elevated red blood cell count (40 cells/μL) but normal white blood cells (4 leukocytes/μL), protein (0.42 mg/dL), and glucose levels (2.8 mmol/L). Initial vasculitis screening (CRP, ANA, C‐ANCA, and P‐ANCA) was unremarkable, and further screening was deferred due to lack of convincing signs or symptoms. Moreover, 21 days following the event, repeat CT brain showed interval improvement of the hydrocephalus, CT cerebral venography showed patent dural sinuses, and CTA redemonstrated multifocal intracranial narrowing with no significant hemodynamic stenosis or occlusion. Brain MRI with time of flight and vessel wall imaging sequences was performed to rule out occult vascular malformation, vasculitis, PRES, and amyloid angiopathy. It showed multifocal stenosis without any corresponding vessel wall T1 hyperintensity or enhancement, which are in keeping with vasospasm. Whole spine MRI showed no evidence of occult vascular malformation or cord pathology. She was discharged 6 days later after significantly improving to fluids and analgesia and an uneventful hospital course. Nine months later, she underwent a diagnostic cerebral angiogram to reassess the previously noted findings, which revealed resolution of the prior cerebral vasculature narrowing (Figure 3).

She was ultimately diagnosed with RCVS, fulfilling the diagnostic criteria outlined in the International Classification of Headache Disorders, Third Edition (ICHD‐3) and achieving an RCVS_2_ score of seven points (five points for presence of thunderclap headache, one point for female sex, and one point for the presence of SAH). In her most recent clinic follow‐up 40 months after the initial incident, she remained in her baseline status with a modified Rankin scale of zero.

## 3. Discussion

This report highlights the neuroradiological characteristics of a rare case of COVID‐19–associated RCVS in a middle‐aged female initially presenting with features of SAH and later showing transient constriction of CTA. In the absence of typical RCVS triggers, such as medication use, comorbidities, trauma, or physical exertion, and with the only notable factor being a cough associated with COVID‐19, we propose that COVID‐19 was an underlying trigger for RCVS.

To establish an association, rather than a mere coincidence, between COVID‐19 and RCVS, it is imperative to exclude alternative etiologies. Vasoactive medications include triptans (used for migraine treatment), serotonergic drugs (including antidepressants), and oral contraceptives have all been associated with RCVS [6]. Additionally, pseudoephedrine, a common component in over‐the‐counter nasal decongestants and cough suppressants often used by patients with COVID‐19, is a vasoconstrictor known to contribute to RCVS [7]. Jung et al. conducted a systematic review on COVID‐19–related RCVS and found that among 24 reported cases, 85% of patients had used medications known to trigger RCVS [5]. They also reported four patients (16.7%) without any known triggers, relevant medical history, or medication use, of whom three did not have a reported cough [5]. It is possible that cough played a role in our case. Kato et al. reported a case of a 52‐year‐old female without a known respiratory illness who presented with a cough‐induced headache, which was later diagnosed as RCVS. They hypothesized that RCVS may have been triggered by increased intracranial pressure caused by coughing, leading to an abnormal sympathetic response [8]. It remains unclear whether SARS‐CoV‐2 directly caused RCVS in our case and other reported cases or whether factors such as coughing or the use of certain medications to manage COVID‐19 contributed to its development. On a cellular level, it is plausible that SARS‐CoV‐2 played a role in triggering RCVS. The infection is associated with the downregulation of ACE‐2 receptors, and both animal and small human studies have shown that this dysregulation of the renin–angiotensin system can lead to vasoconstriction in patients with COVID‐19 [9, 10]. This mechanism has been hypothesized to drive COVID‐19–associated RCVS. The time from COVID‐19 symptom onset to the development of RCVS in our case aligns with the findings in the literature. Bonura et al. conducted a systematic review in 2023 examining 20 patients diagnosed with RCVS in association with COVID‐19 and found that neurologic symptoms of RCVS occurred, on average, 13.8 days after the onset of COVID‐19 diagnosis [11]. Due to the rarity of COVID‐19–associated RCVS, it is challenging to measure the strength of association between the two. Moreover, there is no published experimental evidence in animal models to draw an association between the two.

Thus far, this association may meet the temporality and biological plausibility criteria of the Bradford Hill criteria. These criteria provide a framework which is used to evaluate whether an observed relationship between an exposure and an outcome is causal [12]. However, it is important to recognize that case reports and case series alone are insufficient to establish causality. While meeting some of these criteria may provide a foundation and encourage further investigation and rigorous studies to clarify the relationship between COVID‐19 and RCVS.

In patients presenting with SAH and multifocal arterial narrowing, several alternative etiologies were carefully considered. Primary angiitis of the central nervous system is a possible mimic but is typically supported by inflammatory CSF abnormalities and MRI or vessel wall imaging features of vasculitis and often shows a more persistent or progressive course; in our case, CSF white blood cells and protein were within normal limits, inflammatory screening for systemic vasculitis was unremarkable, vessel wall imaging showed no mural enhancement, and subsequent follow‐up angiography demonstrated resolution of arterial narrowing, which strongly favors a reversible vasoconstrictive process over vasculitis. Inflammatory cerebral amyloid angiopathy was less likely given the patient’s age and the absence of supportive MRI markers such as microbleeds, cortical superficial siderosis, or characteristic inflammatory changes. PRES can rarely present atypically with vasoconstriction, but our patient remained normotensive, and MRI did not demonstrate vasogenic edema or other features consistent with PRES [13]. Diagnosis of RCVS relies on clinical and radiological features. According to the ICHD‐3, a patient must experience a severe headache, with or without neurological symptoms, that is either sudden or triggered by specific activities. The headache should resolve or be ongoing for less than 3 months. Additionally, an angiogram must reveal a “string of beads” appearance in the blood vessels, indicating the characteristic narrowing associated with RCVS [14]. The RCVS‐2 is not only similar to the ICHD‐3 criteria but also includes gender, presence of SAH on imaging, and sparing of intracranial carotid arteries [15]. The diagnosis of RCVS was confirmed after our patient achieved an RCVS2 score of seven points. An RCVS2 score of ≥ 5 demonstrated 90% sensitivity, 99% specificity, and 96% positive predictive value for diagnosing RCVS [15].

Compared with previously reported COVID‐19–associated RCVS cases, several features of our case are notable. First, the initial presentation was dominated by extensive SAH complicated by hydrocephalus requiring emergent EVD placement, showcasing the hemorrhagic severity at onset. While convexity SAH is a recognized complication of RCVS, extensive SAH with hydrocephalus is less commonly noted [16]. Second, our patient had no identifiable typical precipitants, including no exposure to vasoactive or serotonergic medications (including over‐the‐counter decongestants such as pseudoephedrine), oral contraceptives, illicit substances, postpartum state, or exertional triggers. This contrasts with the predominance of medication‐related triggers in prior reports and systematic reviews of COVID‐19–associated RCVS [5]. Third, we provide long longitudinal outcome data, with radiographic confirmation of reversibility on repeat angiography at 9 months and clinical follow‐up to 40 months demonstrating complete functional recovery (mRS 0). This duration of follow‐up exceeds that reported in most prior cases and adds a prognostic value by confirming sustained clinical recovery after angiographic resolution.

## 4. Conclusion

This case report highlights the potential link between COVID‐19 and RCVS, particularly in the absence of typical triggers such as medications, physical exertion, or comorbidities. Our patient presented with thunderclap headache, SAH, and multifocal cerebral vasospasm, in the context of COVID‐19, and was ultimately diagnosed with RCVS after achieving an RCVS_2_ score of 7 points. While a direct association between SARS‐CoV‐2 and RCVS cannot be conclusively established from a single case, the temporal association and underlying biological plausibility suggest that COVID‐19 may contribute to the onset of RCVS. This report contributes to the growing body of evidence that suggests COVID‐19 may have diverse neurological manifestations and should prompt clinicians to consider RCVS in patients with similar presentations. Further research and larger cohort studies are needed to better understand the pathophysiological mechanisms of RCVS in the context of COVID‐19 infection and ascertain association between the two.

## Author Contributions

Seraj Makkawi: conceptualization, writing–original draft, writing–review and editing, and supervision. Osama Khojah: data curation, investigation, writing–original draft, and writing–review and editing. Mohammed Alsalmi: data curation, investigation, writing–original draft, and writing–review and editing. Adilah Alturaifi: data curation, investigation, writing–original draft, and writing–review and editing Bader Ibrahim: data curation, writing–original draft and writing–review and editing Abdulrahman Alshamy: conceptualization, writing–original draft, and writing–review and editing.

## Funding

The authors declare having received no financial support.

## Consent

To maintain anonymity, all identifying information about the patient was removed, and therefore, informed consent was not required for this case report.

## Conflicts of Interest

The authors declare no conflicts of interest.

## Data Availability

The data that support the findings of this study are available from the corresponding author upon reasonable request.
